# Functional Application of Tricks for Super Obese Patient Positioning: A Technical Guide for Hip Fractures on a Fracture Table With a Case Example

**DOI:** 10.7759/cureus.21932

**Published:** 2022-02-05

**Authors:** Nina D Fisher, Andrew S Bi, Noah Kirschner, Abhishek Ganta, Sanjit R Konda

**Affiliations:** 1 Orthopedic Surgery, New York University Langone Medical Center, New York, USA; 2 Orthopedics, New York University Langone Medical Center, New York, USA; 3 Orthopedic Surgery, Jamaica Hospital Medical Center, New York, USA

**Keywords:** intraoperative positioning, technique, elderly, obese, hip fracture

## Abstract

Obese patients with hip fractures are at increased risk of perioperative complications due to both their size and associated medical conditions. The purpose of this report is to describe a technique for intraoperative positioning of obese patients who sustain a hip fracture. A 62-year-old female with a history of morbid obesity (BMI 48.06kg/m^2^), type II diabetes mellitus, and hypertension presented with a right intertrochanteric fracture and was admitted for operative fixation on a fracture table. A standardized approach for systematic patient positioning and abdominal panniculus taping is described, which facilitates operative repair of the hip fracture using a cephalomedullary nail. This report describes the intraoperative positioning technique of a morbidly obese patient with an intertrochanteric hip fracture in order to highlight specific techniques used to deal with the physical aspects of obesity that can improve the surgical efficiency of the procedure. By positioning obese patients in a standardized way, intraoperative time and complications will be decreased, potentially mitigating some of the risks associated with this patient population.

## Introduction

Obesity, defined as body mass index (BMI) greater than 30kg/m^2^, continues to be a growing problem within the United States [1]. Obesity is associated with multiple medical comorbidities and leads to greater rates of perioperative complications and associated costs [2-10]. With respect to orthopedic trauma, obese patients intraoperatively are at greater risk of nerve injuries secondary to positioning, intraoperative complications, increased blood loss, and increased overall operative time [7,11]. Furthermore, the use of a fracture table for operative fixation of hip fractures has its inherent risks related to patient positioning, including neurologic injury, damage to the perineal integument, and well-leg compartment syndrome [11]. Given the elevated risk of perioperative morbidity and mortality, intraoperative techniques should be optimized to minimize surgical time to maximize the likelihood of a positive outcome. This is of particular importance in cases when a fracture table is used for the treatment of hip and femur fractures, as this equipment requires meticulous patient positioning prior to sterile draping to achieve adequate fluoroscopic imaging and access to the starting point for the intramedullary nail. Preoperative positioning becomes essential in cases of morbid obesity, defined as BMI greater than 40kg/m^2^ or greater than 35kg/m^2^ with an obesity-related health condition, as the patient’s central obesity, abdominal panniculus, and excess soft-tissue can significantly impede imaging and implant positioning. This report describes the intraoperative positioning technique of a morbidly obese woman with a right intertrochanteric hip fracture to highlight specific tips and tricks used to deal with the physical aspects of obesity in order to improve the surgical efficiency of the procedure.

## Case presentation

History of present illness

**Figure 1:**
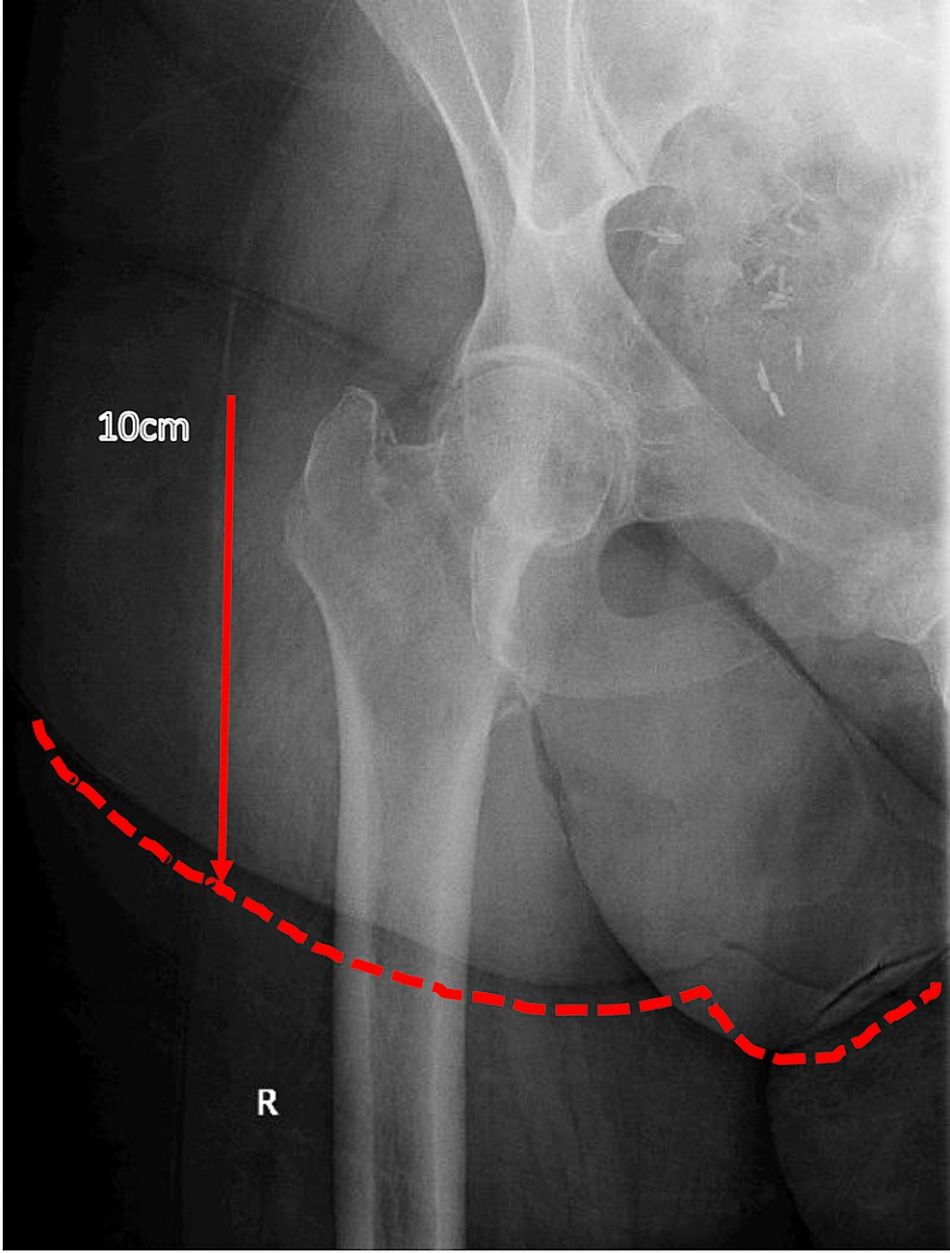
Initial Imaging Anteroposterior radiograph of the right hip demonstrating OTA/AO type 31A1.2 intertrochanteric fracture. The abdominal panniculus fold, extending 10cm distal to the tip of the greater trochanter and outlined in red, is overlying the anterior and lateral aspect of the right hip joint. OTA/AO: Orthopaedic Trauma Association/Arbeitsgemeinschaft für Osteosynthesefragen

The patient was a 62-year-old female with a history of morbid obesity (BMI 48.06kg/m2), type II diabetes mellitus, and hypertension who presented with a right Orthopaedic Trauma Association/Arbeitsgemeinschaft für Osteosynthesefragen (OTA/AO) type 31A1.2 intertrochanteric fracture (Figure 1) and was admitted to the orthopedic service for operative repair.

Physical examination

**Figure 2:**
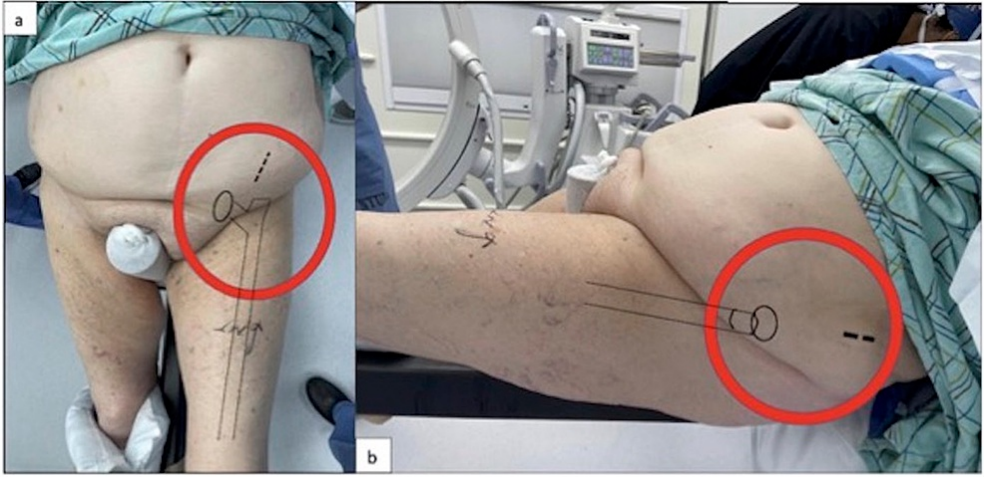
Example of Pannus Representative patient images demonstrating abdominal panniculus overhanging planned surgical site in the frontal (a) and sagittal (b) planes.

The patient was noted to have central obesity with a large abdominal panniculus that overhung her right hip and obscured the planned surgical incision site for intramedullary nail entry into the greater trochanter (Figure 2). The patient also had large pendulous breasts that fell below the midaxillary line that would impede in-line reaming of the intramedullary canal and excessive soft-tissue at both of her proximal thighs that was problematic with lateral imaging of the proximal femur. 

Surgical technique

**Figure 3:**
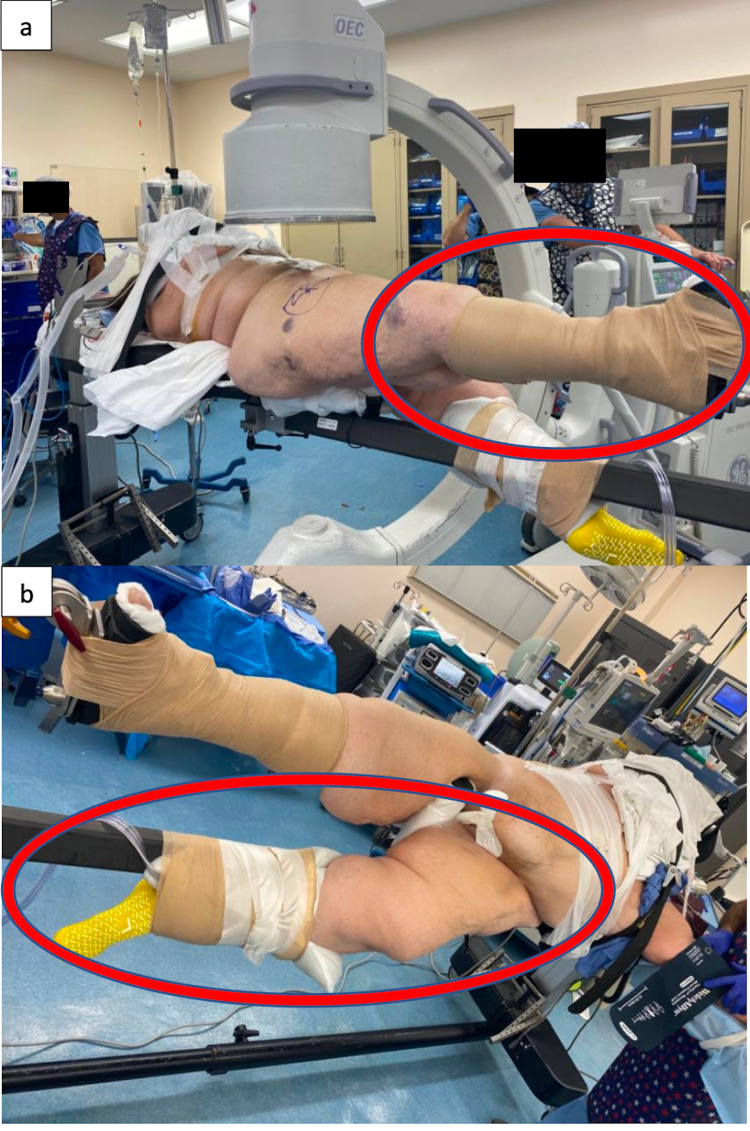
Positioning on Fracture Table a. Patient’s operative extremity is placed in a padded fracture boot and further secured with Coban^TM^. b. Patient’s non-operative extremity is scissored below the level of the operative extremity, and the lower leg is wrapped with a pillow and secured to the fracture table. The non-operative extremity is slightly externally rotated to ensure it is not in line with the operative leg, as doing so may complicate obtaining fluoroscopic images.

Informed consent was obtained from the patient for the use of images related to the surgery. The patient underwent general anesthesia with endotracheal intubation on the hospital bed and was transferred to a fracture table, which was confirmed preoperatively to hold 500 pounds. Immediately, the perineal post was placed to secure the patient on the bed and minimize the risk of falling. Her right lower extremity was positioned in the fracture boot, with all prominences padded and heel maximally seated. Coban^TM^ was extended proximally to the level of the tibial tubercle to provide added control of the leg and traction during the procedure (Figure 3a). The patient’s left lower extremity was scissored below the level of the operative extremity, wrapped with a “pillow taco”, with additional Coban^TM^ used to secure the leg given the excess weight (Figure 3b). The contralateral limb was slightly externally rotated and placed lower to the operative extremity to prevent excess soft tissue at the patient’s proximal thighs from impeding the lateral fluoroscopic images. The patient’s abdominal panniculus, breasts, and ipsilateral upper extremity were then positioned in a systematic manner.

**Figure 4:**
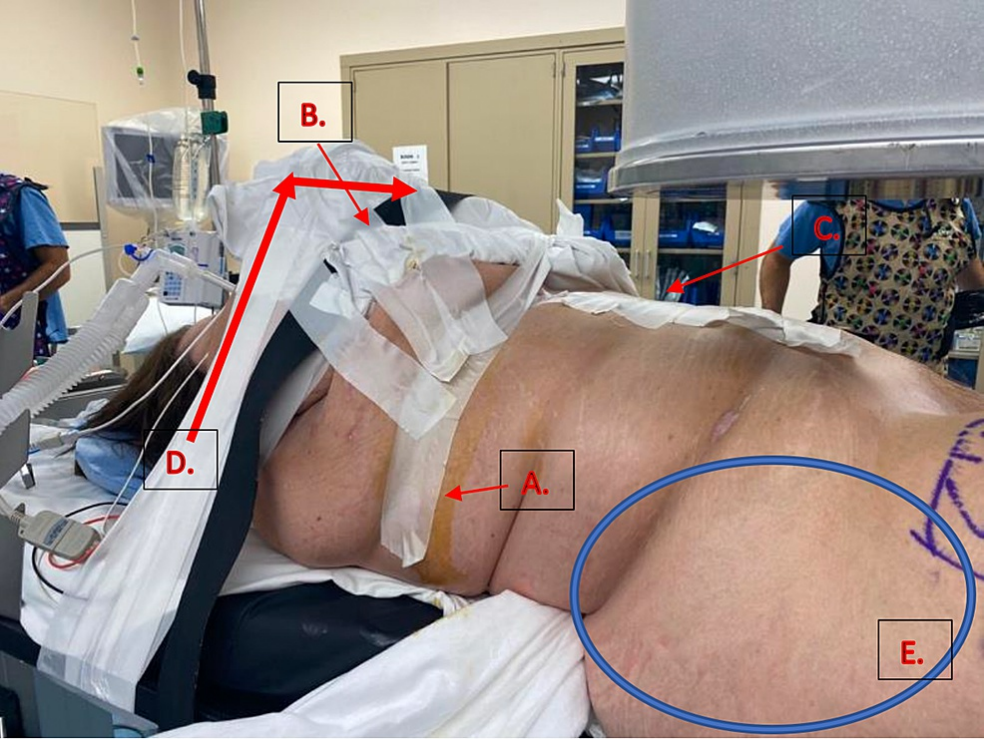
Systematic Abdominal Panniculus Taping A. Mastisol® is used to create a sticky surface on the skin that is also protective against skin tears. Surgical tape (silk) is then placed on the Mastisol® to create a “landing strip” to which additional tape can adhere for different vectors of pull. B. Tape in this orientation is used to pull the patient’s ipsilateral breast proximally out of the surgical field. C. Tape is placed on the patient’s midline and extended towards the opposite side and secured to the table in order to pull the central abdominal panniculus and remainder of the mid-torso excess soft tissue away from the patient’s midline. D. The patient’s ipsilateral arm is placed over her chest, pulled towards the contralateral side and taped to the bed to rotate the upper torso towards the patient’s contralateral side. E. Note the wide exposure of the proximal hip that is free of overhanging central abdominal panniculus and soft tissue that allows for improved access to the starting point for the intramedullary nail (compared to Figure 5C) where the contralateral hip is completely obscured by the patient’s central abdominal panniculus and mid-torso soft tissue.

First, a layer of Mastisol® liquid adhesive was placed just below both of the patient’s breasts on her abdomen, running from midline to the midaxillary line. Mastisol® creates a surface more suitable for taping and protects against skin tears, an important risk to consider when using significant amounts of tape on bare skin for patient positioning. A layer of silk tape was then placed over the area of Mastisol® (Figure 4) to create a “landing zone” or anchorage point for multiple sites of strategic taping during subsequent steps.

Small strips of tape were placed on the landing zone in a vertical fashion to pull the patient’s ipsilateral breast proximally out of the surgical field (Figure 4). These strips also assist in pulling excess soft tissue in the axilla and mid-torso proximally, thereby minimizing excess soft tissue in the surgical field that would impede access to the start point. 

Another layer of Mastisol® was placed in the midline of the patient’s abdomen, and a vertically oriented piece of silk tape was placed. Multiple strips of tape were applied transversely from the midline on the abdomen toward the contralateral side in order to sequentially place tension on the patient’s central abdominal panniculus to pull it away from the surgical field (Figure 4). These horizontally oriented strips are made long enough to be secured to the contralateral side of the table for maximal soft tissue retraction. Placing the tape in this fashion pulls the central pannus as well as the remainder of the mid-torso’s excess soft tissue towards the contralateral side in order to improve the exposure of the surgical starting point.

**Figure 5:**
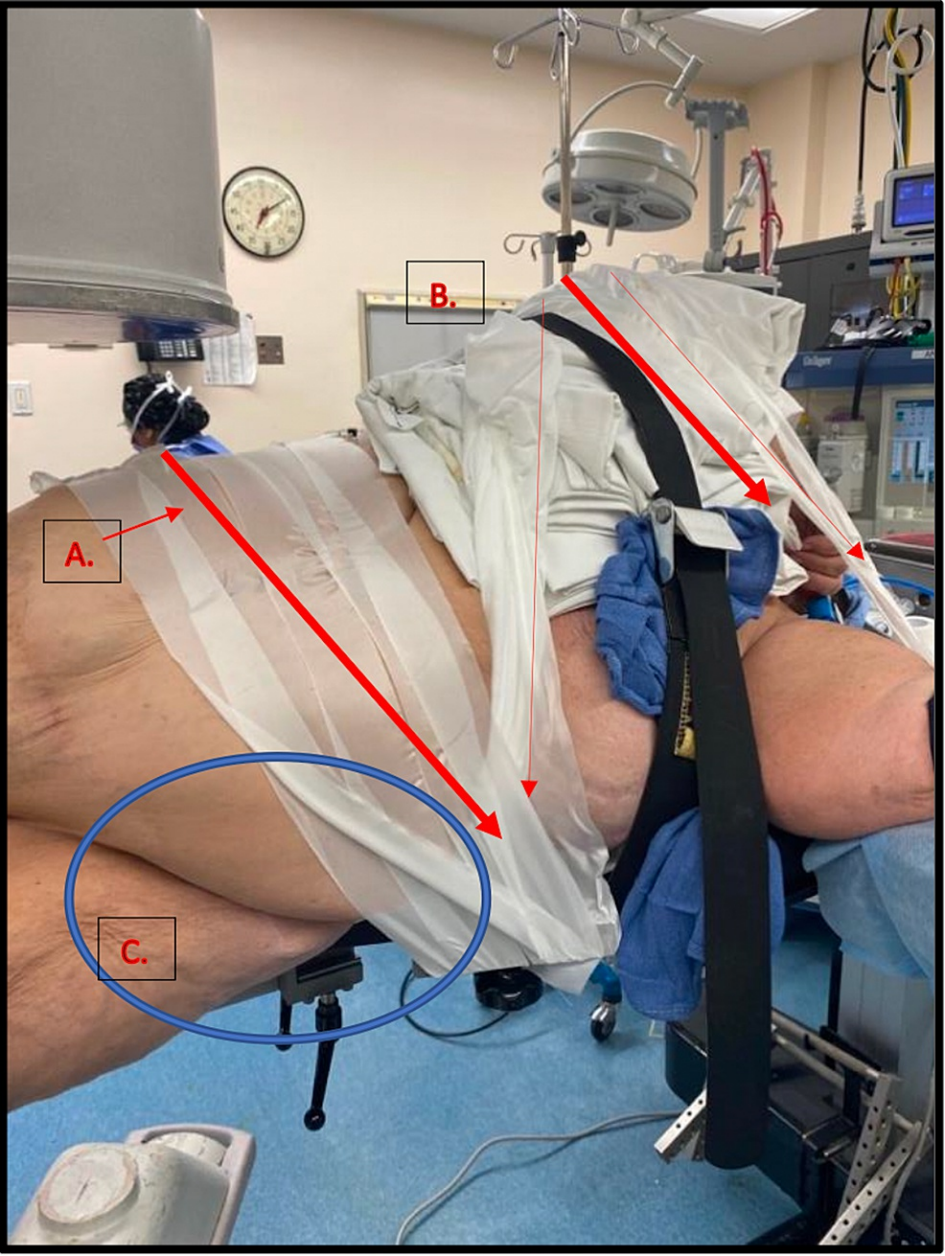
Contralateral Side A. Horizontal strips of silk tape are secured to the patient’s abdominal panniculus, pulled towards the contralateral side and secured to the table. B. Ipsilateral arm positioned over the patient’s chest with silk tape used to secure the arm. This same tape is also pulling the patient’s ipsilateral breast away from the surgical field. C. Note the abundance of abdominal panniculus that is overhanging the patient’s contralateral hip completely obscuring the operative site at the proximal hip precluding insertion of an intramedullary nail.

Finally, the patient’s ipsilateral arm was placed over her chest and secured to the opposite side of the bed (Figure 5). The arm was secured first with tape, covered with significant padding, then further secured with a large safety strap (Figure 5). For patients with significant central obesity, it is important to ensure that the torso is securely positioned, as the weight of the torso can cause the patient to shift significantly during surgery, which may not be recognized immediately by surgical staff. Taping the ipsilateral limb across the body also serves to rotate the upper torso to the patient’s contralateral side, further assisting in pulling excess soft tissue away from the surgical field.

Figure 4 demonstrates the final result of systematic abdominal panniculus taping: a surgical field free of overlying or obstructing soft tissue, allowing for access to the appropriate surgical starting point. Additionally, the soft tissue is positioned in a way that will not interfere with reaming during the surgical procedure itself. Comparatively, Figure 5 demonstrates the abundance of abdominal panniculus on the patient’s contralateral side that would prevent the insertion of an intramedullary nail.

**Figure 6:**
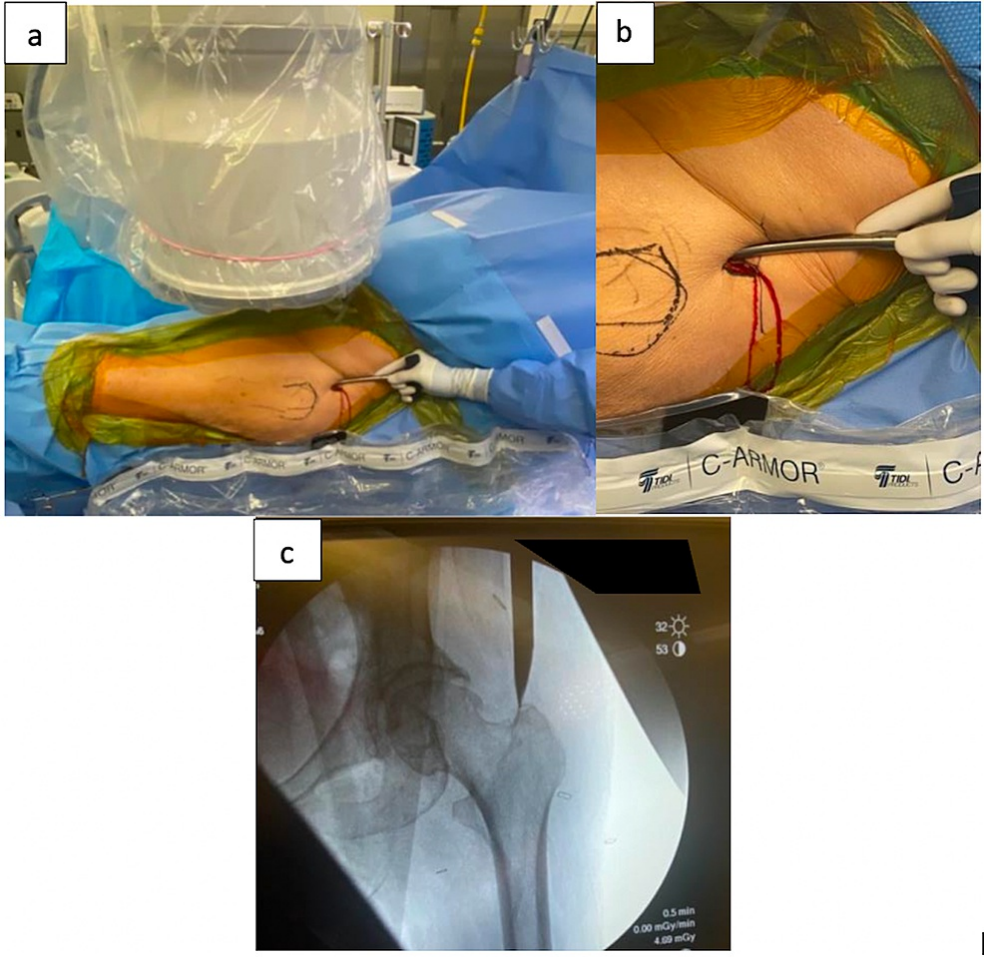
Start Point with Awl A curved cannulated awl (a) is used to obtain the start point, allowing for penetration of subcutaneous tissue and medial pressure against the lateral soft tissues (b) to maintain control of the tip of the awl (c).

**Figure 7:**
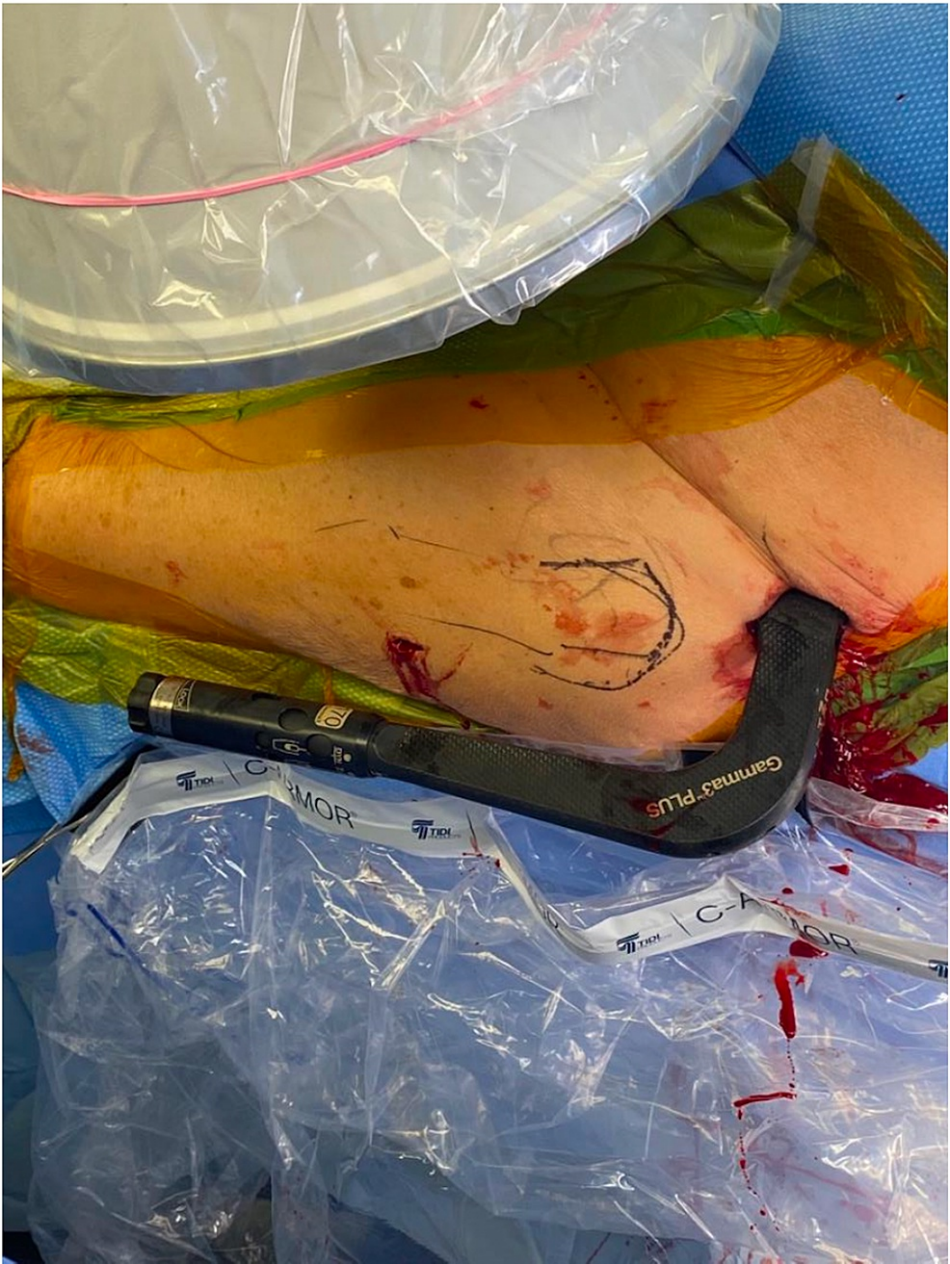
Lateral Excursion Jig A percutaneous jig with extra lateral excursion is used for percutaneous lag screw placement to avoid impingement of soft tissues on the jig.

**Figure 8:**
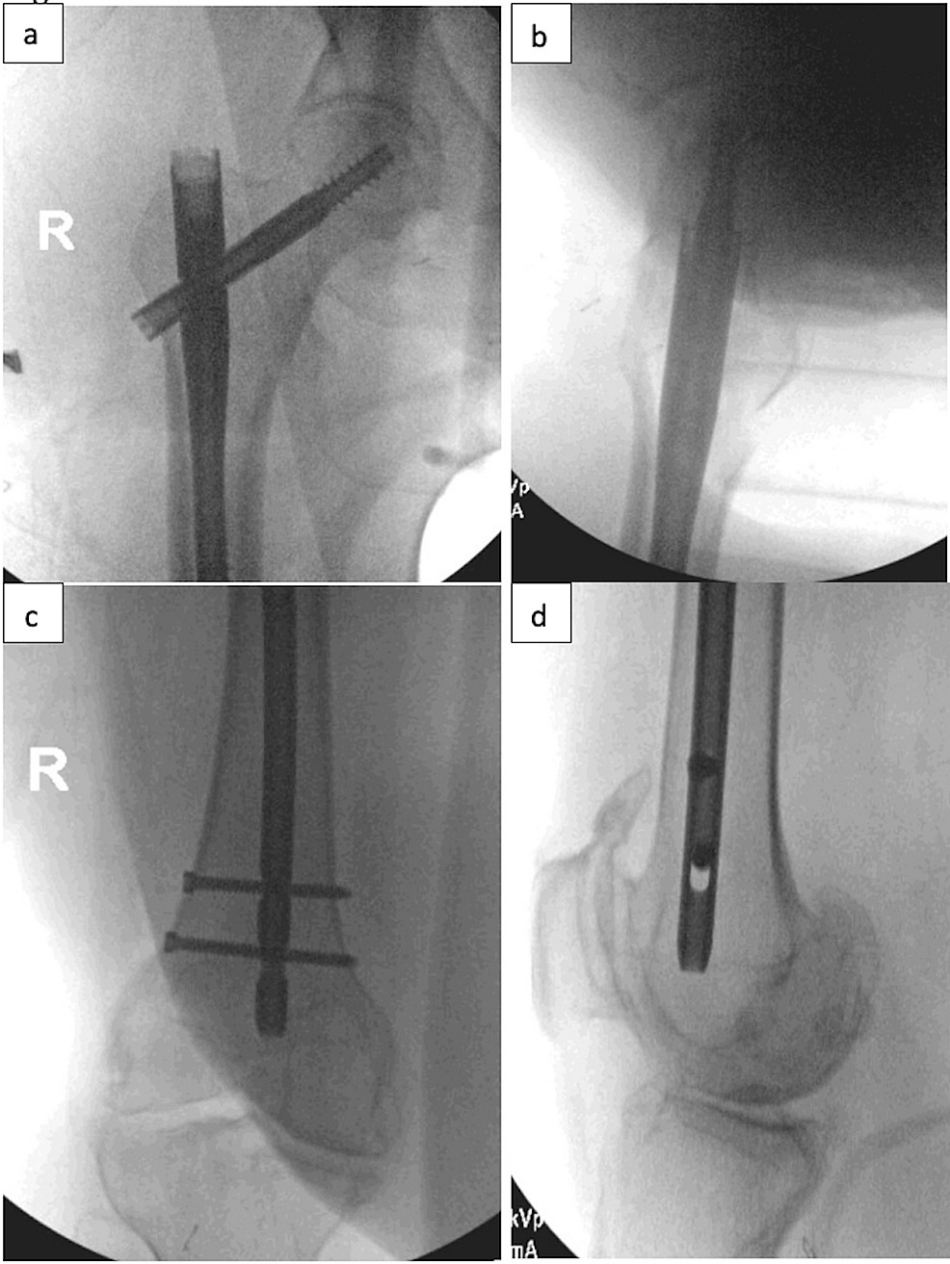
Final Fluoroscopy Final fluoroscopic images demonstrate acceptable fracture reduction and implant position.

The right intertrochanteric fracture was reduced under fluoroscopy on the fracture table, and the right lower extremity was subsequently prepped and draped. An additional prep stick was used to ensure that the entire exposed surface area was appropriately prepped. Surgical landmarks were identified, although with difficulty given the amount of soft tissue between the bones and skin, and a 5 cm longitudinal incision was made proximal to the greater trochanter. A cannulated curved awl was inserted to determine the greater trochanteric start point given the distance from skin to start point (Figure 6). In obese patients, the awl is able to better penetrate the abundant subcutaneous tissue and allow for medial pressure against the abdominal panniculus and other soft tissues while maintaining control of the tip of the awl. With a guidewire alone, there is often deflection of the wire against the abdominal panniculus, making it difficult to control the tip of the guidewire. Once the start point was established, the starting wire was inserted through the awl and secured at the starting point before reaming with the opening reamer. A long cephalomedullary device was placed in an uncomplicated fashion with the use of a percutaneous jig with extra lateral excursion, which allows for percutaneous lag screw placement while avoiding impingement of the buttocks and proximal thigh soft tissues on the jig (Figure 7). If there is soft tissue impingement on the jig, it can lead to deviation in the trajectory of the guidewire into the femoral head, leading to suboptimal lag screw placement and associated increased risk of hardware failure. Two distal interlocking screws were placed in a static position using the perfect-circle technique. Final fluoroscopic images demonstrate acceptable reduction and hardware position (Figure 8). Total surgical time, including anesthesia and positioning, was 121 minutes, with operative time from incision to closure of 54 minutes.

Postoperative course

Postoperatively, the patient returned to a regular room and was started on deep vein thrombosis (DVT) prophylaxis. She was ultimately discharged to subacute rehabilitation on postoperative day six. At one year follow-up, she was ambulating with an assistive device with minimal pain, and radiographs demonstrated a healed intertrochanteric fracture with no evidence of hardware complication.

## Discussion

Despite the high complication rate seen in the obese patient population, there is limited recommendation on perioperative strategies to optimize care for morbidly obese patients in the setting of orthopedic trauma surgery. Bozzio et al., in a review article, recommended retracting soft tissues, skin folds, and abdominal panniculus with paper tape in order to optimize exposure for appropriate surgical access [12]. Mulcahey et al., in an early study, reviewed perioperative management of obese patients [13]. While prior studies have reviewed perioperative management of obese patients and use of paper tape for abdominal panniculus retraction, to the authors’ knowledge, there are no other studies that describe intraoperative positioning and draping for morbidly obese patients in order to achieve appropriate exposure, and none specifically reporting on a fracture table with patient examples [12,13]. This method will serve as a standardized method of positioning obese patients who sustain hip fractures to improve their care.

As obesity becomes a more common diagnosis within the hip fracture patient population, it becomes necessary to develop a standardized intraoperative approach to positioning to care for this specific patient population to avoid the known increased risk of complications [14-17]. A recent study by Bekeris et al. reported an increased incidence of comorbidities, including obesity amongst hip fracture patients, and Sems et al. reported increased complication rates following surgical fixation of pelvic ring injuries in obese patients [14,15]. Furthermore, a study of 950 patients by Muller et al. reported that increased BMI in patients with hip fractures is associated with both longer operation time and length of hospitalization [16]. Specifically, intertrochanteric hip fracture patients with a BMI greater than 30kg/m^2^ are more likely to sustain systemic complications, including respiratory complications, electrolyte abnormalities, and sepsis [18]. Rosenfeld et al. reported on the difficulties in spine surgery when positioning morbidly obese patients, especially with prone positioning [19]. All obese patients do not fare equally, and it is often the morbidly obese, defined as those with a BMI greater than 40kg/m^2^, who experience the greatest risk of complications [18,20]. Ditillo et al. demonstrated in a large retrospective analysis of the National Trauma Data Bank that across all adult trauma patients, morbid obesity is associated with increased in-hospital complication rates, longer hospital and intensive care unit stay, and higher rates of mortality [17]. Thus, it becomes prudent for orthopedic surgeons to mitigate intraoperative risks as much as possible by standardizing patient positioning.

## Conclusions

The purpose of describing this technique is to optimize the surgical care of morbidly obese patients with hip fractures to decrease intraoperative time and thus reduce the associated complications of prolonged anesthesia, blood loss, and positioning. By positioning obese patients in an expedited and standardized way, surgeons will facilitate access to the operative site and perform the operation with less difficulty, thereby decreasing operative time, which may decrease complications in this patient population. We hope this report guides future practice in positioning morbidly obese patients with hip fractures using a fracture table.
